# Discovery of a new *Hepatozoon* species namely *Hepatozoon viperoi* sp. nov. in nose-horned vipers in Türkiye

**DOI:** 10.1038/s41598-023-36814-w

**Published:** 2023-06-15

**Authors:** Onur Ceylan, Letícia Pereira Úngari, Gonca Sönmez, Cigdem Gul, Ceylan Ceylan, Murat Tosunoglu, Bengi Baycan, Lucia Helena O’Dwyer, Ferda Sevinc

**Affiliations:** 1 Department of Veterinary Parasitology, Faculty of Veterinary Medicine, Selcuk University, 42130 Konya, Türkiye; 2 Instituto de Biociências, Campus de Botucatu, Departamento de Parasitologia, UNESP-Univ. Estadual Paulista, Distrito de Rubião Junior, Botucatu, SP 18618-689 Brazil; 3 Department of Genetics, Faculty of Veterinary Medicine, Selcuk University, 42130 Konya, Türkiye; 4 Department of Biology, Faculty of Sciences, Çanakkale Onsekiz Mart University, Çanakkale, Türkiye; 5 Department of Parasitology, Faculty of Veterinary Medicine, Siirt University, 56100 Siirt, Türkiye; 6 Department of Biology, School of Graduate Studies, Çanakkale Onsekiz Mart University, Çanakkale, Türkiye

**Keywords:** Genetics, Microbiology, Zoology, Diseases, Pathogenesis

## Abstract

Although *Hepatozoon* spp. remains the most prevalent intracellular protozoa infecting snakes, it was reported only in a few snake species of the Colubridae family in Türkiye. Moreover, studies on these hemoparasites are not available in venomous nose-horned vipers from Türkiye. In this study, we investigated *Hepatozoon* spp. in three individual *Vipera ammodytes* using morphological and molecular methods. Our results were positive for intraerythrocytic *Hepatozoon* spp. gamonts in all three snakes, exhibiting low parasitemia. The microscopic findings were further confirmed through molecular data. A genus-specific PCR assay targeting the 18S rRNA gene region of *Hepatozoon* spp., was performed using HemoF/HemoR and Hep300/Hep900 primers. The obtained sequences were concatenated and used in phylogenetic analyses in comparison with different *Hepatozoon* species. Although our (OP377741) isolate was separated into a different branch, it was clustered with the isolates of *H. massardi* (KC342526), *H. cevapii* (KC342525), and *H. annulatum* (ON262426) from Brazilian snakes. Moreover, gene similarity and pair-wise distance between our isolate and other *Hepatozoon* species infecting snakes were found to be 89.30–98.63% and 0.009–0.077, respectively. Hence, we reported a new species of *Hepatozoon*, namely *Hepatozoon viperoi* sp. nov. infecting *V. ammodytes*. Since the literature does not indicate the existence of such a *Hepatozoon* species in *V. ammodytes* in different countries, our data may contribute to the expanding knowledge of *Hepatozoon* species in snakes, providing new insights into the biodiversity of the haemogregarine protozoan parasite.

## Introduction

Haemogregarine protozoa belonging to the *Hepatozoon* genus (Miller, 1908) (Adeleorina: Hepatozoidae) have a broad host range like birds, lizards, crocodiles, snakes, and mammals, including domestic and wild canids and felids^1^. These protozoa infect the blood cells of various endothermic and ectothermic intermediate vertebrate hosts and lead an obligatory heteroxenous life. The definitive invertebrate hosts, including ticks, mites, fleas, sucking lice, reduvid bugs, phlebotomine flies, anopheline and culicine mosquitoes, and leeches, play a significant role in maintaining their heteroxenous survival^1–3^. Among haemogregarine parasites, *Hepatozoon* is the most prevalent genus infecting snakes^2,3^. Contrary to the mode of transmission in many vector-borne diseases, *Hepatozooon* infection is usually transmitted horizontally in snakes via oral ingestion of infected invertebrates or intermediate prey^4^.

In a broad spectrum of animals, particularly mammals and reptiles, as well as birds and amphibians, hepatozoonosis is characterized by moderate to severe symptoms, including anemia, cachexia, fever, lethargy, hyperglobulinemia, weight loss, and anorexia^5,6^. Although *Hepatozoon* species are believed not to cause clinical disease due to their excellent host adaptation, many cells (liver, lung, kidney, spleen) can still be damaged during their asexual reproduction. However, in the case of severe infections, hemolytic anemia can also be observed. Furthermore, dehydration, lethargy, open-mouth breathing, and weight loss may be encountered in immune-compromised and aberrant hosts^7^.

More than 340 *Hepatozoon* species cause diseases in animals having veterinary importance, including domestic and wild animals^8^. Previously, new *Hepatozoon* species or isolates have been detected in different countries in various snake species through advanced molecular diagnostic techniques^9–13^. However, many of them were related to the *Hepatozoon* species in colubrid snakes, with restricted information available on viper snakes. Studies are particularly scarce on the snakes belonging to the *Vipera* genus of the Viperidae family. Also, some studies have not shown the expected results^14,15^. Although some *Hepatozoon* species were detected in various animals in Türkiye^6^, one study in particular reported *Hepatozoon* infections in colubrid snakes^15^. However, the investigation of hemogregarine protozoans has not been done at the microscopic or molecular level in nose-horned vipers i.e., *Vipera ammodytes* in Türkiye.

Thus, the main objective of this study was to detect the presence of *Hepatozoon* species in nose-horned vipers and determine their phylogenetic relationships with various species or isolates of other snakes and reptiles using morphological examination and molecular techniques.

## Material and methods

### Study animals

**Figure 1:**
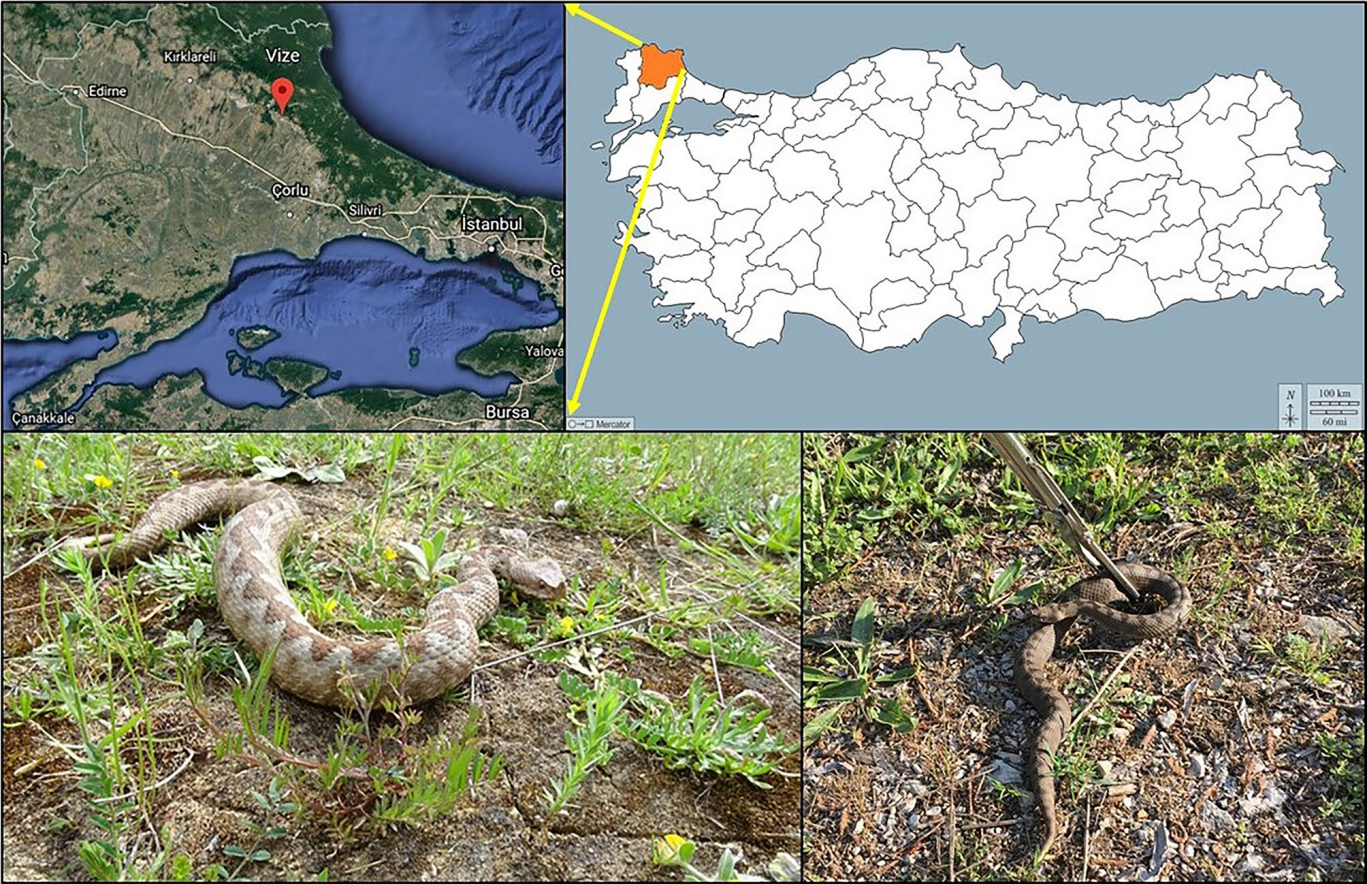
The Vize district of Kırklareli province in Türkiye where the nose-horned viper specimens were captured (Satellite image and map were obtained from https://earth.google.com and https://d-maps.com/carte.php?num_car=25320&lang=en, respectively)^16^.

The study consisted of three female nose-horned vipers (*Vipera ammodytes* Linnaeus, 1758) captured in 2022 from the Göztepe locality (41° 35′ 3.71″ N, 27° 47′ 48.70″ E) of the Vize district of Kırklareli province, Türkiye. Nose-horned vipers are venomous species and generally live in stony and rocky areas. For this study, the vipers were captured using a snake grab stick. Figure 1 shows the locality from where these nose-horned vipers were captured.

### Blood samples and microscopic examination

The nose-horned vipers were firstly restrained to collect blood. Then, approximately 2 mL of blood was collected through the post-orbital sinus in heparinized hematocrit tubes^17,18^. Next, the blood samples were transferred to a tube containing ethylenediaminetetraacetic acid (EDTA), and thin blood smears were prepared from each blood sample. The samples were then air dried, fixed in absolute methanol for 5 min, and stained for 20 min using Wright’s staining method^19^. After smear preparation, the remaining blood samples were stored at − 20 °C for further molecular analysis while the captured horned vipers were released back to their habitats. All smears were examined under a light microscope (Olympus CX 31) at 100× magnification. Relevant literature was used to identify the gamont stages of intraerythrocytic parasites^3^.

### DNA extraction

Blood samples of each nose-horned viper were used to extract genomic DNA using a commercial DNA extraction kit (E.Z.N.A.^®^ Blood DNA Mini Kit, Omega BIO-TEK, Georgia, USA), which was performed following the manufacturer’s instructions. The concentrations of the eluted DNA were measured using a nanodrop spectrophotometer (Colibri, TITERTEK BERTHOLD) while the genomic DNAs were stored at − 20 °C for further molecular analysis. Since we did not have enough blood for DNA extraction from one of the vipers, we used the blood and DNA samples from the other two vipers to extract genomic DNA and perform further processes.

### PCR amplification and pathogen detection

We performed two PCR assays targeting two different regions of the 18S rRNA gene of apicomplexan parasites by using two primer sets. The HemoF/HemoR primers were used to amplify the 900 bp region while Hep300/Hep900 was used to amplify 600 bp of the 18S rRNA gene region of *Hepatozoon* spp.^20,21^. PCR was performed by making minor modifications to the PCR thermal cycles. Table 1 lists the oligonucleotide sequences used in the PCR assay to detect *Hepatozoon* spp. The reaction mixture for each PCR assay consisted of 10 µL of ExPrime Taq Premix (2X, No dye), 0.2 µL of forward primer, 0.2 µL of reverse primer, 4 µL of DNA template, and 5.6 µL of double-distilled water, making up the final volume to 20 µL. BioRad thermocycler was used for amplification. The PCR programs for the two different primer sets were as follows: HemoF/HemoR; 95 °C for 5 min (initial denaturation), 35 cycles of (94 °C for 30 s, 48.8 °C for 30 s, 72 °C for 1 min), and 72 °C for 5 min (final extension), Hep300/Hep900; 94 °C for 3 min (initial denaturation), 35 cycles of (94 °C for 45 s, 56 °C for 1 min, 72 °C for 1 min) and 72 °C for 7 min (final extension). Distilled water was used for the negative control. The amplified DNA samples were electrophoresed on the agarose gel (1%) in TAE buffer. After electrophoresis, the gel was stained with ethidium bromide and photographed using a UV transilluminator (UVP, Upland, CA, USA).

**Table 1:** Primer pairs used in the PCR assay to detect *Hepatozoon* spp.

Oligonucleotides	Sequences	Target gene	Expected size (bp)	Reference no
HemoF/HemoR	HemoF: 5′TATTGGTTTTAAGAACTAATTTTATGATTG-3′ HemoR: 5′CTTCTCCTTCCTTTAAGTGATAAGGTTCAC-3′	18S rRNA	900 bp	^19^
Hep300/Hep900	Hep300: 5′-GTTTCTGACCTATCAGCTTTCGACG-3′ Hep900: 5′-CAAATCTAAGAATTTCACCTCTGAC-3′	18S rRNA	600 bp	^20^

### Sanger sequencing and phylogenetic analysis

PCR products were sequenced in both directions by a commercial company. The obtained sequence chromatograms (forward and reverse sequences) were assembled, and each primer sequence (HepF300/900 and HemoF/R) was edited out to obtain a partial 18S rRNA consensus sequence. These sequences were concatenated (~ 1200 bp) using the Geneious version 7.1.3 (Bomatters, “http://www.geneious.comww”)^22^. Also, the sequences belonging to the haemogregarine group in Genbank were aligned using Geneious version 7.1.3 by the MUSCLE algorithm (Bomatters, “http://www.geneious.comww”)^22^. Following Úngari et al.’s study, *Adelina dimidiata* Schneider, 1875, *Adelina grylli* Butaeva, 1996 and *Klossiella equi* Baumann, 1946 were selected as outgroups^23^. Phylogenetic relationships were inferred by the Bayesian inference (BI) and Maximum likelihood (ML) analysis through MrBayes 3.2.2 and RAxML 7.2.8., respectively^24,25^. For ML method, JModelTest v.2.1.10 was used to identify the best evolutionary model^26^. Based on Akaike information criterion (AIC) the Transitional model with a discrete Gamma distribution (TVM + G) was chosen^27^. Phylogenetic analysis was inferred using PhyML with 1000 replicate bootstraps (> 50%)^28^. The BI analysis was carried out using MrBayes implemented from the computational resource CIPRES^29^, the best BIC score was the General Time Reversible model (GTR + I + Γ)^30^. The Markov chain Monte Carlo (MCMC) algorithm was run for 10,000,000 generations, sampling one tree every 1000 generations. On the burn-in, the first 25% of generations were discarded, and the consensus trees were estimated using the remaining trees. Bayesian posterior probabilities (BPP) cut off was considered > 50%. The aligned sequences of haemogregarine species infecting snakes were compared using a pair-wise distance (p-distance) matrix.

### Accession number status

We submitted the sequences obtained from two different primer sets (HemoR/F, Hep300/900) targeting different regions of the 18S rRNA gene region to the GenBank, National Center for Biotechnology Information with the following accession numbers: ON619569/OM866261 (VA1), OM866262/OM866263 (VA2) [HemoR/HemoR], and ON673853/ON673854 (VA1), ON629811/ON629812 (VA2) [Hep300/Hep900]. However, since these sequences represented a short part of the target gene region, they were not included in the phylogenetic analyses. Instead, contig sequences amplified using both primer pairs were concatenated to obtain a sequence representing a longer part (~ 1200 bp) of the 18S rRNA gene (OP377741), which was then used in phylogenetic analyses (https://www.ncbi.nlm.nih.gov/nuccore/OP377741.1/).

### Statistical analysis

The experimental data were analyzed using the SPSS 25 program (IBM Corp. Released 2017. IBM SPSS Statistics for Windows, Version 25.0. Armonk, NY: IBM Corp.). Data were evaluated after controlling for normal distribution prerequisites (Kolmogorov–Smirnov test). Since the differences between the two independent groups did not meet the parametric test prerequisites, the Mann–Whitney U test was used. Variables were expressed as median (min/max) values. The values of *p* < 0.05, *p* < 0.01, and *p* < 0.001 were accepted as the significance levels of the tests.

### Ethics approval

All experimental protocols were approved by Ethics Committee of Çanakkale Onsekiz Mart University (Approval number: HADYEK, 2021/10–05). All methods were carried out in accordance with relevant guidelines and regulations and in compliance with the Animal Research: Reporting of In Vivo Experiments (ARRIVE) guidelines. After collecting blood samples, horned vipers were released back into the areas from which they were captured.

## Results

We found that all three nose-horned vipers (*V. ammodytes*) in the Thrace Region of Türkiye were infected with the *Hepatozoon* species. The infected vipers were adult females with low parasitemia. We observed mature gamonts of *Hepatozoon* in the erythrocytes of the hosts. Since we did not perform histopathological examinations, we could not evaluate other developmental stages of the parasite. The molecular and phylogenetic analysis verified the species as an undescribed *Hepatozoon* spp. in *V. ammodytes*.

### Species description

Phylum: Apicomplexa Levine, 1970.

Class: Conoidasida Levine, 1988.

Subclass: Coccidia Leucart, 1879.

Order: Eucoccidiorida Leger, 1911.

Suborder: Adeleorina Leger 1911.

Family: Hepatozoidae Wenyon, 1926.

Genus: *Hepatozoon* Miller, 1908.

Species: *Hepatozoon viperoi* Ceylan, Ungari and Sevinc sp. nov.

### Taxonomic summary

*Type-host*: nose-horned viper *Vipera ammodytes* Linnaeus, 1758 (Serpentes: Viperidae: Viperinae).

*Type-locality*: Göztepe locality (41° 35′ 3.71″ N, 27° 47′ 48.70″ E) in Vize district of Kırklareli province in Türkiye.

*Site of infection*: erythrocytes (blood).

*Vector*: unknown.

*Etymology*: considering the genus name of the infected host (*Vipera ammodytes*), the protozoan was named *Hepatozoon viperoi* sp. nov. This is the first *Hepatozoon* species reported in the nose-horned viper belonging to the *Vipera* genus.

*Parasitemia*: 0.1–0.4%.

*Deposited materials*: blood smears and DNA samples from *V. ammodytes*. These materials are deposited in Selcuk University, Faculty of Veterinary Medicine, Department of Veterinary Parasitology.

*Gene sequence*: the 18S rRNA concatenated gene sequence obtained from the blood of *V. ammodytes* was submitted to GenBank under the accession number (OP377741).

### Microscopy

**Figure 2:**
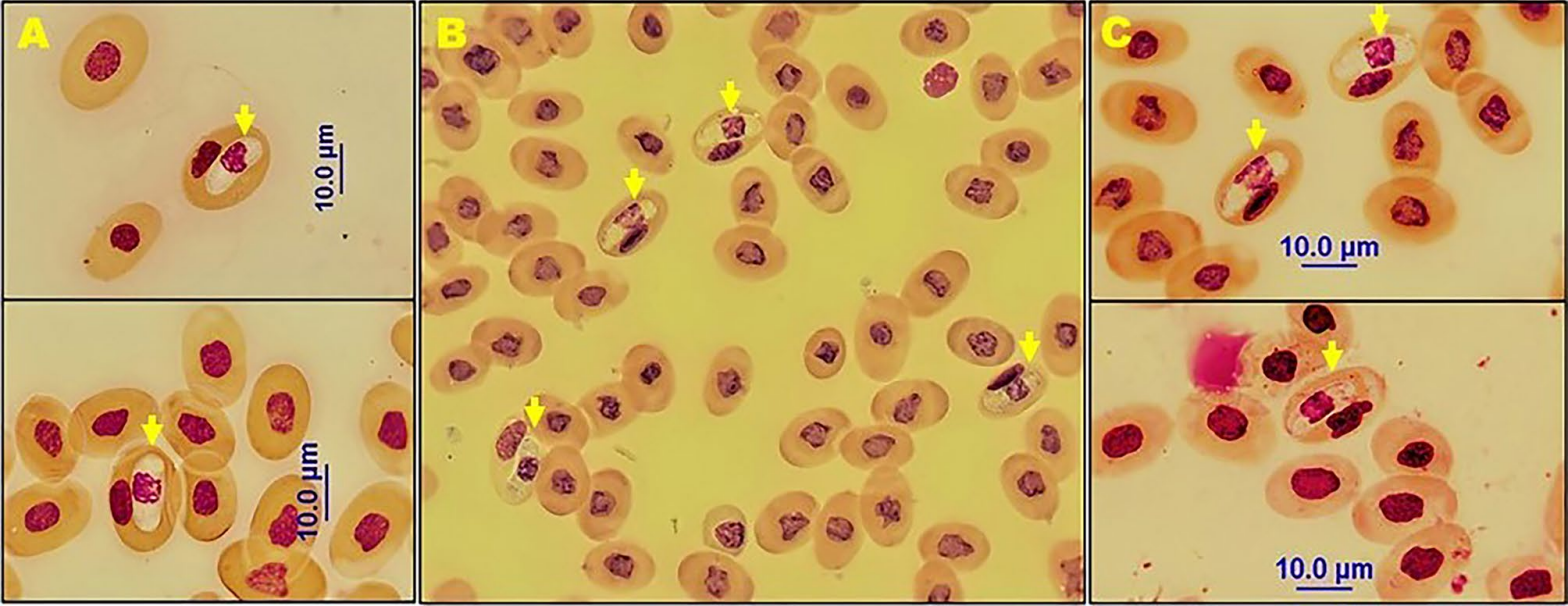
Photomicrographs of intraerythrocytic stages of *Hepatozoon viperoi* sp. nov. Miller, 1908 found in blood samples of *Vipera ammodytes*; intraerythrocytic gamonts of *Hepatozoon viperoi* sp. nov. showing the centric nucleus and no vacuoles [**A**: VA1 (×100 magnification); **B**: VA2 (×40 magnification); **C**: VA3 (×100 magnification)].

We found that the nose-horned viper *V. ammodytes* was infected with a *Hepatozoon* species. In this study, gamonts of *Hepatozoon* spp. were detected microscopically in thin blood smears of all captured horned vipers. Figure 2 shows different microscopic images of gamonts in each snake, separately. Although the parasitemia level was quite low (< 0.1%) in VA1 and VA3 samples, it was slightly higher in VA2 (0.4%) sample. Overall, our morphological and morphometric data along with the molecular and phylogenetic analysis revealed a new *Hepatozoon* species.

### Morphological and morphometric analysis of gamonts

Gamonts of the *Hepatozoon viperoi* sp. nov. were detected only in the erythrocytes of *V. ammodytes*, with no other developmental stages being observed in the thin blood smears. Gamonts were enclosed in a thin parasitophorous vacuole. They were elongated and had one end more tapered while the cytoplasm was stained whitish pink. Sausage-shaped gamonts were found near the nucleus of the infected erythrocytes, exhibiting a uniform cytoplasm. Furthermore, gamonts displaced the nucleus of the host erythrocytes. Table 2 presents the morphometric parameters including the length and width of the erythrocytes and the length and width of the nucleus of the gamonts. The pinkish-purple gamont nuclei were centrally located and were mostly quadrangular in shape. The length, width, and area of the gamonts (mean ± standard deviation) were 14.05 ± 0.44 µm, 5.83 ± 0.42 µm (n = 15), and 64.96 ± 6.64 µm^2^, respectively. The length, width, and area of the nucleus of the parasite were 6.35 ± 0.65 µm, 5.20 ± 0.47 µm, and 22.91 ± 3.25 µm^2^ (n = 20), respectively.

**Table 2:** Morphometric parameters of the gamonts of *Hepatozoon viperoi* sp. nov. and the host cells of nose-horned viper (*Vipera ammodytes*).

Feature	Mean	S.D	Range
**Mature gamonts**			
Length	14.05 µm	0.44 µm	13.28–14.93 µm
Width	5.83 µm	0.42 µm	4.89–6.44 µm
Length of nucleus	6.35 µm	0.65 µm	5.59–7.98 µm
Width of nucleus	5.20 µm	0.47 µm	4.11–5.78 µm
Gamont area	64.96 µm^2^	6.64 µm^2^	54.37–77.78 µm^2^
Nuclei area	22.91 µm^2^	3.25 µm^2^	17.38–29.82 µm^2^
**Uninfected erythrocyte**			
Length	15.60 µm	0.69 µm	14.06–16.67 µm
Width	11.22 µm	0.66 µm	10.19–12.47 µm
Length of nucleus	6.92 µm	0.72 µm	5.89–8.47 µm
Width of nucleus	5.50 µm	0.49 µm	4.84–6.34 µm
Erythrocyte area	144.83 µm^2^	17.11 µm^2^	117.50–173.43 µm^2^
Nuclei area	27.98 µm^2^	6.70 µm^2^	15.84–45.68 µm^2^
**Infected erythrocyte**			
Length	18.31 µm	0.71 µm	16.90–19.89 µm
Width	11.91 µm	0.88 µm	11.25–14.33 µm
Length of nucleus	8.80 µm	1.05 µm	7.15–10.86 µm
Width of nucleus	3.93 µm	0.70 µm	2.96–5.55 µm
Erythrocyte area	165.07 µm^2^	11.17 µm^2^	138.98–187.03 µm^2^
Nuclei area	22.79 µm^2^	5.74 µm^2^	11.02–34.06 µm^2^

Except for the area, the sample size was 15 for all other measurements (n = 20).

The nucleus of the infected erythrocyte had an elongated appearance and was compressed between the gamont and the membrane of the erythrocyte. The infected host erythrocytes showed significant hypertrophy (*P* < 0.05) along with higher mean length (*P* < 0.001) and width (*P* < 0.05). Although the areas of infected erythrocytes were significantly higher than the areas of non-infected erythrocytes (*P* < 0.001), the area of their nuclei remained smaller (*P* < 0.05). The length, width, and area of the infected erythrocytes were 18.31 ± 0.71 µm, 11.91 ± 0.88 µm (n = 15), and 165.07 ± 11.17 µm^2^ (n = 20), respectively, while the length, width, and area of the uninfected erythrocytes were 15.60 ± 0.69 µm, 11.22 ± 0.66 µm (n = 15), and 144.83 ± 17.11 µm^2^ (n = 20), respectively. Table 2 indicates the detailed measurement values of the morphometric parameters.

### Remarks

Although the literature review reported on the presence of *Hepatozoon* spp. in some snakes of Türkiye, no *Hepatozoon* species have been formally described (using morphological or a combined approach of morphology and molecular) in snakes so far. Tome et al. detected *Hepatozoon* spp. at the molecular level in some colubrid snakes, including *Dolichophis caspius*, *Elaphe sauromates,* and *Natrix tessellata*, however, the isolates were not identified at the species level^15^. This said, no morphological or morphometric data on *Hepatozoon* species are available in snakes in Türkiye. The morphological and morphometric characteristics of *Hepatozoon viperoi* sp. nov. determined in our study can be compared to *Hepatozoon* species found in some Brazilian snakes, which may show a phylogenetic relationship. To date, although no studies have reported the occurrence of *Hepatozoon* species in *V. ammodytes*, some new species including *H. cuestensis*, *H. cevapii,* and *H. massardi* have been described in rattlesnakes (*Crotalus durissus terrificus*). These rattlesnakes belong to the genus Crotalus, subfamily Crotalinae, and family Viperidae. Among these, *H. cevapii* and *H. massardi* showed phylogenetic proximity to *H. viperoi* sp. nov. described in our study. Therefore, we used these two species for the morphological and morphometric comparison. Moreover, we included a recently described new species *H. annulatum,* found in a Brazilian colubrid snake *Leptodeira annulata*, in our comparison due to its phylogenetic proximity to *H. viperoi* sp. nov.

The morphology of gamonts of all aforementioned *Hepatozoon* species, including *H. viperoi* sp. nov., caused the displacement of erythrocyte nuclei. All species of gamonts appeared to be elongated, with one end slightly more tapered than the other. The cytoplasmic structures of the *H. cevapii* and *H. massardi* gamonts showed basophilic and granulous cytoplasm. However, the cytoplasm of *H. viperoi* sp. nov. gamonts showed uniformity. Differentiating *H. viperoi* sp. nov. from the other species based on microscopic examination may be difficult due to their similar morphology. However, at this point, morphometric measurements can act as a guide.

The length, width, and area of the *H. cevapii* gamonts found in the colubrid snake species *Oxyrhopus rhombifer* in Brazil were 14.81 ± 0.99 µm, 5.02 ± 0.76 µm, and 64.38 ± 4.70 µm^2^, respectively, while the length, width, and area of the parasites’ nucleus were 4.35 ± 0.28 µm, 3.99 ± 0.98 µm, and 14.01 ± 1.87 µm^2^ (n = 30), respectively^13^. The area of the *H. cevapii* gamonts was similar to that of the *H. viperoi* sp. nov., with minor differences in other morphometric measurements. However, significant morphometric differences were observed between the gamonts’ nuclei. The values of the nuclei length, width, and area of *H. viperoi* sp. nov. gamonts were higher than that of *H. cevapii*.

The length, width, and area of the *H. massardi* gamonts found in *Crotalus durissus terrificus* species in Brazil were 17.4 ± 0.7 µm, 3.0 ± 0.3 µm, and 38.9 ± 3.7 µm^2^, respectively, while the length, width, and the area of the parasites’ nucleus were 4.7 ± 0.3 µm, 2.3 ± 0.2 µm, and 9.1 ± 0.8 µm^2^, respectively^9^. Although the gamont length of *H. viperoi* sp. nov. was shorter than that of *H. massardi*, the other morphometric measurements including the gamont width and area and the nucleus length, width, and area were higher in the gamonts of *H. viperoi* sp. nov.

The length, width, and area of *H. annulatum* gamonts described in *Leptodeira annulata*, a colubrid snake species in Brazil, were 14.25 ± 0.54 µm, 5.34 ± 0.26 µm, and 64.32 ± 5.90 µm^2^, respectively, while the length, width, and the area of the parasites’ nucleus were 3.91 ± 0.63 µm, 4.13 ± 0.29 µm, and 16.95 ± 2.01 µm^2^, respectively^13^. The morphometric measurements of the *H. viperoi* sp. nov. including the gamont’s length, width, and area were found to be very similar to that of *H. annulatum*. However, the measurements of the nucleus of the gamont showed significant differences between both species.

*Hepatozoon. viperoi* sp. nov. detected in our study showed phylogenetic similarity with *H. cevapii*, *H. massardi,* and *H. annulatum* species found in various Brazilian snakes. However, these three *Hepatozoon* species have not been detected in Türkiye yet. The absence of morphological-morphometric studies of *Hepatozoon* spp. in the snakes of Türkiye led us to compare our findings with those found in the snakes of other countries. Although morphological examination plays a significant role in the differential diagnosis, it is still difficult to predict the *Hepatozoon* species without morphometric measurements, which requires further molecular confirmation.

### Molecular data, sequencing, and phylogenetic analysis

Genomic DNAs were amplified using two sets of primers (HemoF/HemoR, Hep300/Hep900) targeting different regions of the 18S rRNA gene of *Hepatozoon*. Amplified products were sent for sequencing, and the obtained sequences were submitted to the GenBank database of the National Center for Biotechnology Information. The contig sequences obtained using the pair of primers HemoF and HemoR were further aligned and compared using the Geneious version 7.1.3 (Bomatters, www.geneious.comww)^22^. Since these sequences were 100% similar, another pair of primers (HepF300/900) was used to amplify another part of the 18S rRNA gene, which was then concatenated with the sequence amplified using HemoF/HemoR resulting in a ~ 1200 bp sequence (OP377741).

**Figure 3:**
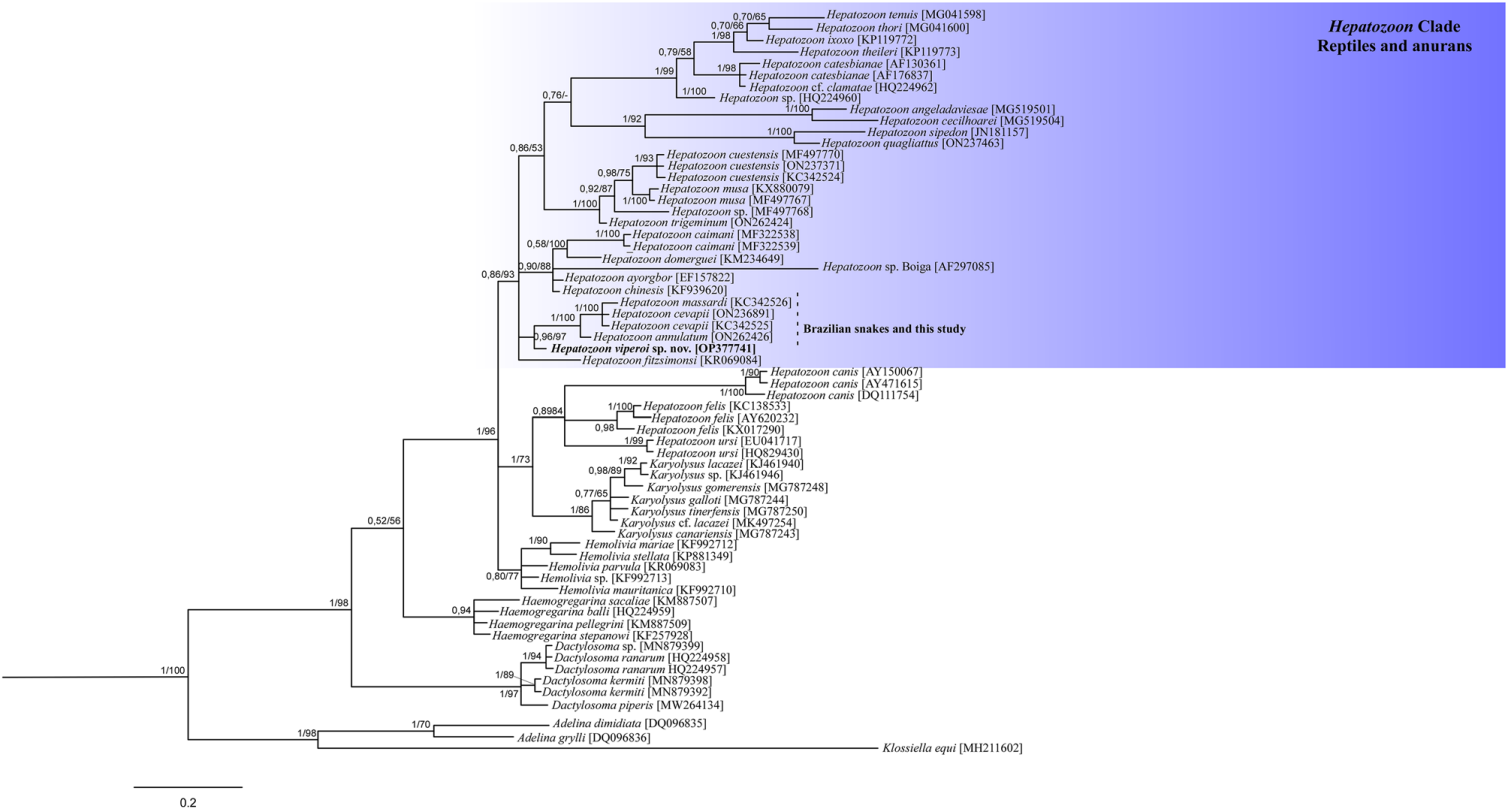
Consensus phylogram of haemogregarines based on 18S rDNA sequences. The topology trees with Bayesian inference (BI) and Maximum likelihood (ML) analyses were identical (represented by the BI tree). The values associtaed with the branches are related to the bootstrap values (BI/ML). The scale bar represents 0.02 nucleotide substitutions per site. *Adelina dimidiata* (DQ096835), *Adelina grylli* (DQ096836) and *Klossia equi* (MH211602) were used as outgroups.

Both Bayesian Inference (BI) and the Maximum Likelihood (ML) phylogenetic analyses resulted in identical tree topologies (Fig. 3). The phylogenetic analyses included isolates of adeleorinid parasites (Haemogregarinidae, Hepatozoidae, Karyolysidae, and Dactylosomatidae) available in GenBank. *Hepatozoon* spp. belong to polyphyletic groups that form two separate clades according to their vertebrate host species. *Hepatozoon* spp. isolated from large mammals form a sister clade with the *Karyolysus* genus while the isolates obtained in our study clustered with the sequences of the reptile and anuran hosts belonging to a large Hepatozoidae clade. Although our isolate clustered with the isolates obtained from Brazilian snakes, including *Hepatozoon massardi* O´Dwyer, Moço, Paduan, Spenassatto, Silva and Ribolla 2013 (KC342526), *Hepatozoon cevapii* O´Dwyer, Moço, Paduan, Spenassatto, Silva and Ribolla 2013 (KC342525/ON236891) and *Hepatozoon annulatum* Úngari, Netherlands, Silva and O´Dwyer 2022 (ON262426), it was clustered on a different branch. The gene similarity and pair-wise distance are summarized in Table 3.

**Table 3:** Gene similarity and pair-wise distance between *Hepatozoon viperoi* sp. nov. and other *Hepatozoon* isolates infecting various snakes found in the Genbank.

*Hepatozoon* from snakes	Gene similarity (%)	Pair-wise distance
**1.** OP377741—*H. viperoi* sp. nov		
**2.** KC342525—*H. cevapii*	98.40	0.012
**3.** KC342526—*H. massardi*	98.18	0.014
**4.** MF497770—*H. cuestensis*	97.50	0.019
**5.** ON237463—*H. quagliattus*	93.18	0.066
**6.** ON262426—*H. annulatum*	98.40	0.014
**7.** ON262424—*H. trigeminum*	97.95	0.009
**8.** KX880079—*H. musa*	97.04	0.024
**9.** AF297085—*H*. sp. Boiga	89.30	0.061
**10.** MG519501—*H. angeladaviesae*	92.95	0.072
**11.** MG519504—*H. cecilhoarei*	92.50	0.077
**12.** JN181157—*H. sipedon*	92.27	0.074
**13.** EF157822—*H. ayorgbor*	98.63	0.009
**14.** KF939620—*H. chinensis*	98.40	0.012

## Discussion

The snake species *Vipera ammodytes* (Linnaeus, 1758) is characterized by its prominent fleshy horn at the tip of the snout. As one of the most venomous vipers in Eurasia, *V. ammodytes* are widely distributed between Austria and Italy, and across the Balkan Peninsula to Türkiye and Caucasus^31,32^. These vipers live in mesophytic and xerophytic forests, dry cliffs, mosaic meadows, screes, scrubs, and even on artificial stone walls. They commonly prey on lizards and small mammals by subduing them with their venom^32,33^. Telford stated that the infection of the snakes with *Hepatozoon* species may be based on the predation of infected vertebrate hosts or the ingestion of the invertebrate vectors^3^. Therefore, in this study, we investigated the existence of *Hepatozoon* spp. in vipers. The microscopic findings revealed the intraerythrocytic sausage-shaped gamonts. However, since microscopy was inadequate for species identification, we further diagnosed it molecularly^34^.

A wide variety of pathogens can infect reptiles, with some groups of parasites receiving little attention. As a result, their biodiversity awaits clarification. Systematics of parasites commonly rely on some features, including the morphological and lifecycle characteristics of parasites, some of which are difficult to evaluate. However, molecular techniques have eliminated these difficulties, in turn, increasing the knowledge of their biology, biodiversity, and phylogeny^34–36^. In this study, we determined the presence of *Hepatozoon viperoi* sp. nov. in *Vipera ammodytes* both microscopically and molecularly. Although many such species have been identified in snakes, our study may further contribute to the information on biodiversity and phylogeny of the *Hepatozoon* genus, which is believed to have not received enough attention. Studies on new *Hepatozoon* species or isolates in snakes are contributing to the study of haemogregarine protozoan fauna^9–11,37^. A literature review indicated the inadequacy of studies on haemoprotozoan infections in reptiles in Türkiye, especially snakes. A study conducted by Tome et al. investigated *Hepatozoon* species in some colubrid snakes, namely *Coronella austriaca* (Smooth snake), *Dolichophis caspius* (Caspian whipsnake), *Eirenis modestus* (Dwarf snake), *Elaphe sauromates* (Blotched rat snake), *Hemorrhois nummifer* (Coin-marked snake), and *Natrix tessellata* (Tessellated water snake)^15^. Additionally, *Hepatozoon* spp. were detected in *D. caspius* (KJ408513) in Kırklareli, *E. sauromates* (KJ408514) in Erzurum, and *N. tessellata* (KJ408526) in Karakose. *Vipera* genus along with colubrid snakes is an essential member of the Turkish herpetofauna, especially in the Thrace region and the parts of Eastern Anatolia close to Georgia and Armenia^31^. The available literature does not have data on the morphological or molecular detection of *Hepatozoon* spp. in snakes belonging to the Viperidae family in Türkiye. Therefore, the originality of the present study is based on the lack of studies detecting *Hepatozoon* spp. in *Vipera* species of different countries.

A study conducted by Tome et al. in Portugal reported no causative agent in snub-nosed vipers, *Vipera latastei* (Bosca) (n = 22)^15^. Similarly, even in Poland, *Hepatozoon* spp. was not detected in the European adder *Vipera berus* (n = 48)^14^. Nasiri et al. conducted a study on parasites in Iranian snakes and investigated three species from the *Vipera* genus but found no blood parasites in the zigzag mountain vipers (*Vipera albicornuta*) and Transcaucasian meadow vipers (*Vipera ursinii eriwanensis*)^38^. Although they observed intraerythrocytic gametocytes of hemoparasites in West-Asian blunt-nosed vipers (*Vipera lebetina obtusa*), they did not perform any molecular confirmation at the species or genus level. The data remains insufficient on the molecular or microscopic prevalence of *Hepatozoon* spp. in vipers belonging to the genus *Vipera*, however, these parasites were detected in several viper species belonging to other genera of the Viperidae family^9,14,15,37–42^. Previously, in Brazil, O’Dwyer et al. detected *Hepatozoon cevapii* sp. nov., *Hepatozoon cuestensis* sp. nov., and *Hepatozoon massardi* sp. nov. in highly venomous pit vipers and *Crotalus durissus terrificus* (rattlesnake)^9^. In a similar study, Ungari et al. reported *H. musa* and *H. cuestensis* in *Crotalus durissus* of Brazil^37^. In Morocco and Mauritania, Tome et al. detected *Hepatozoon* spp. in Saharan horned vipers (*Cerastes cerastes*) while Medrano-Tupiza et al. detected *H. seurati* in the same snake species in Egypt^15,41^. In Saudi Arabia, *Hepatozoon bashtari* n. sp. was detected in *Echis coloratus*, which is a painted saw-scaled viper^42^, while in Pakistan and Egypt, *Hepatozoon echisi* and *H. mehlhorni* were both reported in the species *Echis carinatus*, respectively^43,44^. In this study, for the first time, the presence of *Hepatozoon* spp. was investigated in *V. ammodytes*, and a new *Hepatozoon* species, *Hepatozoon viperoi* sp. nov., was identified both microscopically and molecularly. Sequencing and phylogenetic analysis revealed that our concatenated sequence (OP377741) clustered with sequences from the reptile and anuran hosts belonging to a large Hepatozoidae clade. *Hepatozoon viperoi* sp. nov. clustered with other isolates, including *Hepatozoon massardi* O´Dwyer, Moço, Paduan, Spenassatto, Silva and Ribolla 2013 (KC342526), *Hepatozoon cevapii* O´Dwyer, Moço, Paduan, Spenassatto, Silva and Ribolla 2013 (KC342525/ON236891), and *Hepatozoon annulatum* Úngari, Netherlands, Silva and O´Dwyer 2022 (ON262426) obtained from Brazilian snakes but on a different branch in the phylogenetic tree. Blastn analysis revealed gene similarity between *Hepatozoon viperoi* sp. nov. and the isolates obtained from snakes of Brazil (93.18–98.40%, PD: 0.009–0.077), Australia (89.30%, PD: 0.061), Canada (92.27%, PD: 0.074), China (98.40%, PD: 0.012), and South Africa (92.50–92.95%, PD: 0.072–0.077). Clustering of our concatenated sequence with the sequences from the reptile and anuran host revealed a relationship between the similarities in the detected nucleotides and the feeding or hunting patterns of nose-horned vipers. Tome et al. stated that the genetic lineages infecting the prey may also parasitize snakes^4^. Our study findings also supported this view, however, further studies encompassing a wider sample of vipers may be required to establish clearer genetic proximities between snakes and prey.

*Vipera ammodytes* are among the strictly protected fauna species mentioned in Annex II of the Bern Convention on the Conservation of European Wildlife and Natural Habitats^45^. Also, recently, it has been listed in the Least Concern (LC) category according to the International Union for Conservation of Nature (IUCN) criteria^46^. Detailed investigations are necessary on whether *Hepatozoon* species or other infectious disease agents could affect various physiological activities of vipers along with exploring the extent of the parasitic effect on them since vipers hold an important place in terms of the biodiversity of Türkiye's herpetofauna. Because diseases occurring in natural populations can adversely affect their chances of survival, mainly the reproductive functioning of living beings, it is crucial to take necessary protective actions. One of the main constraints in studying parasites remains the limited comparative genetic data of different host species, especially, wild populations belonging to various geographical areas. Our study reported a new *Hepatozoon* species in nose-horned viper *V. ammodytes*, which has not been studied previously. Hence, this study can contribute to the lack of literature in this regard.

Wildlife species include unique clues allowing early detection of changes in the ecosystem, including habitat fragmentation, environmental pollution, and the presence of pathogens. Wildlife has been frequently considered as sentinels for pathogen emergence and persistence^47–50^. Therefore, studies regarding the haemoparasites of snakes, a significant part of wildlife, becomes important. In conclusion, we used morphological and molecular techniques to describe a new *Hepatozoon* species (*Hepatozoon viperoi* sp. nov.) infecting *V. ammodytes* found in the Thrace region of Türkiye. To the best of our knowledge, this is the first study reporting a haemoprotozoan parasite in nose-horned viper in Türkiye. The identification of a new *Hepatozoon* species may increase the knowledge of parasitic fauna infecting nose-horned viper and also their biodiversity, which has been poorly studied. Therefore, we believe that this study may help identify unknown threat factors, which in turn, may be required for generating conservation strategies for nose-horned viper.

## Data Availability

All data generated or analyzed during the current study are included in this article. The concatenated sequence has been deposited in the NCBI GenBank database under the accession number OP377741 (https://www.ncbi.nlm.nih.gov/nuccore/OP377741.1/).
